# Plasma kynurenine/tryptophan ratio as a sensitive blood-based diagnostic tool for tuberculosis: a diagnostic accuracy study among children living with HIV

**DOI:** 10.3389/fmicb.2026.1898324

**Published:** 2026-08-25

**Authors:** Clement G. Adu-Gyamfi, Lillian Makhathini, Santiago C. Longlax, Alexandra Portillo Varela, Karine Scheuermaier, Ntsiki Sikhondze, Kennedy Otwombe, Andrew Anthony Adjei, Neil Martinson, Alex Kay, Andrew R. DiNardo, Anna M. Mandalakas, Melinda Suchard

**Affiliations:** 1 Centre for Vaccines and Immunology, National Institute for Communicable Diseases, a Division of the National Health Laboratory Service, Johannesburg, South Africa; 2 Brain Function Research Group, Department of Physiology, School of Biomedical Sciences, Faculty of Health Sciences, University of the Witwatersrand, Johannesburg, South Africa; 3 Global TB Program, Department of Pediatrics, Baylor College of Medicine, Houston, TX, United States; 4 Baylor College of Medicine, Houston, TX, United States; 5 Baylor College of Medicine Children’s Foundation Eswatini, Mbabane, Eswatini; 6 Perinatal Health Research Unit (PHRU), Soweto Matlosana Collaborating Centre for HIV/AIDS and TB, Faculty of Health Sciences, University of The Witwatersrand, Johannesburg, South Africa; 7 Department of Pathology, University of Ghana Medical School, Korle-Bu Teaching Hospital, Accra, Ghana; 8 Global TB Program, Department of Pediatrics, Johns Hopkins University Centre for TB Research, Baltimore, MD, United States; 9 Department of Infectious Diseases, Doherty Institute, University of Melbourne, Melbourne, VIC, Australia; 10 Faculty of Health Sciences, University of the Witwatersrand, Johannesburg, South Africa

**Keywords:** diagnostic accuracy, IDO, K/T ratio, pediatric TB, specificity, target product profile

## Abstract

**Background:**

Pediatric tuberculosis (TB) is challenging to diagnose and confirm. The World Health Organization has prioritized development of non-sputum-based diagnostics. We investigated the role of plasma K/T ratio for TB diagnosis among children with and without HIV.

**Methodology:**

Plasma tryptophan and kynurenine concentrations measured via enzyme-linked immunosorbent assay (ELISA) were used to calculate K/T ratios in a nested case-control design with an optimization cohort (the Lung Cohort Study (LCS)) and a validation cohort (the NIH Stool4TB. The discovery cohort included children with incident TB (*n* = 23) and age- and sex-matched controls (*n* = 75). The Stool4TB validation cohort included participants with and without HIV who had microbiologically confirmed TB (*n* = 15) or clinically diagnosed TB (*n* = 20), and controls (*n* = 25).

**Results:**

LCS participants with HIV-associated TB had higher plasma K/T ratio (median 0.511 [IQR 0.182–0.820]) than controls (median 0.053 [IQR 0.030–0.072]), *p* < 0.0001. Among participants with microbiologically confirmed TB living with HIV, plasma K/T ratio gave a sensitivity of 95% (CI 79–99), specificity of 85% (CI 0.75–0.91), PPV of 85%, NPV of 98%, and AUC of 0.96 (95% CI 0.93–0.99). In the validation cohort, among HIV uninfected children, applying a cut-off value of 0.090 gave a sensitivity of 83 % (CI 0.43–0.99), specificity of 96% (CI 0.80–0.99), PPV of 96%, NPV of 90%, and AUC of 0.92 (95% CI 0.82–1) for laboratory confirmed TB. In the LCS, participants who had received intermittent preventative therapy had lower K/T ratio compared to those who did not (*p* = 0.0191).

**Conclusion:**

Plasma K/T ratio is a sensitive non-sputum-based test for diagnosing TB in children and has promising application within pediatric TB diagnostic algorithms.

## 1 Introduction

Tuberculosis (TB) remains a leading cause of illness and death among children worldwide. In 2024, the World Health Organization (WHO) reported approximately 1.8 million new TB cases, of which ∼11% occurred in children (World Health Organization [WHO], 2024). Pediatric TB remains challenging to diagnose because of limited detection methods and the lack of child-specific biomarkers. Of contemporary TB diagnostics, none offer sufficient sensitivity for clinical use in pediatric patients because children often cannot produce high-quality respiratory samples, tend to have low sputum bacterial loads, or present with extrapulmonary disease or nonspecific clinical or radiographic signs (Basile et al., 2022). As a result, it is estimated that about two-thirds of TB cases in children go undiagnosed (Detjen et al., 2015; Nicol et al., 2011; World Health Organization [WHO], 2022). This significant diagnostic gap and the high mortality rate among untreated children emphasize the urgent need to improve TB detection in children (Dodd et al., 2017). The WHO emphasizes the need for a point-of-care, non-sputum-based test capable of detecting all forms of TB as a key step toward achieving TB control goals (World Health Organization [WHO], 2014, 2015).

Recent improvements in specimen collection methods and the development of rapid molecular diagnostics have enhanced the ability to quickly confirm a diagnosis, enabling prompt initiation of TB treatment. However, although the Xpert MTB/RIF assay has increased case detection compared to culture, it still has relatively low sensitivity in children, with more than half of children receiving TB treatment lacking a microbiologic confirmatory test (Kay et al., 2020; Marcy et al., 2016). A meta-analysis in children comparing Xpert MTB/RIF with culture using induced sputum with TB reported a diagnostic sensitivity of 65% for a single specimen (Detjen et al., 2015), with an increased yield of up to 76% with two sequential specimens. Studies evaluating stool and urine samples for TB case detection among children demonstrate an additional yield of ∼25–50% among adolescents with clinically confirmed TB compared to diagnosis with respiratory specimens (Carratalà-Castro et al., 2025; Kay et al., 2024).

Despite increased efforts to implement new TB diagnostics, very little attention has been given to the enzyme indoleamine 2,3-dioxygenase-1 (IDO1) as a potential host-derived TB biomarker in children. IDO is the rate-limiting enzyme in the pathway that converts tryptophan into kynurenines used for synthesis of nicotinamide (Vitamin B3) (Takikawa et al., 1988). Several targeted metabolomics and transcriptomic analyses of samples from children with TB has identified Increased tryptophan metabolism along the kynurenine pathway (Collins et al., 2020; Dutta et al., 2020; Gjøen et al., 2017; Magdalena et al., 2022; Tornheim et al., 2020). We and others have highlighted increased IDO activity measured as kynurenine-to-tryptophan ratio during active TB in people living with HIV (Adu-Gyamfi et al., 2017, 2020, 2021; Collins et al., 2026; Suzuki et al., 2013; Tornheim et al., 2022). There are a handful of metabolomic studies in children suggesting elevated kynurenine and reduced tryptophan metabolites in Tuberculosis (Dutta et al., 2020) however the potential of plasma K/T ratio as a TB biomarker for the diagnosis of TB in children has received little attention (Tornheim et al., 2022). The K/T ratio offers the benefits of being quantitative and blood-based, with potential to monitor changes over time such as during incident, latent or incipient TB and during TB therapy and preventative therapy. In this study, we evaluated the potential of plasma K/T ratio as a blood-based biomarker for diagnosing active TB in children in a TB and HIV endemic setting.

## 2 Materials and methods

### 2.1 Study participants

Participants included in the analysis were selected from two well-defined pediatric cohorts gathered for TB-HIV-focused studies: The Lung Cohort (LCS) study in Johannesburg, South Africa, and the NIH Stool4TB pediatric cohort established in Mbabane, Eswatini (Kay et al., 2024), both of which had sufficient residual blood samples available for secondary analysis.

#### 2.1.1 The lung cohort study (LCS), Johannesburg, South Africa optimization cohort

The LCS was established by the Perinatal HIV Research Unit (PHRU) at the University of the Witwatersrand in Johannesburg. The LCS enrolled children and adults living with HIV from 2008 to 2016 in Soweto, Johannesburg, South Africa. A total of 754 participants were enrolled and followed for 4 years to monitor lung health events over time. Active TB cases included those with a positive Mtb culture result or those who were smear-positive for acid-fast bacilli (AFB), while a probable TB case was a patient who was smear-positive for AFB with a negative or unknown culture result. A possible TB case was a patient with clinical symptoms and chest radiograph findings consistent with active TB but negative or unconfirmed sputum smear and culture results. Gene Xpert testing was not routine in 2008 to 2016. At the time, Antiretroviral therapy (ART) was initiated according to routine clinical care, with a threshold CD4 count of 200 cells/μL as the criteria for starting ART. Blood, urine, and sputum samples were collected at each visit, and blood samples were stored at −70 °C at the Clinical Laboratory Storage in Johannesburg. For this nested, retrospective study, we evaluated all incident pediatric TB cases with available plasma samples and clinical data. Using a 1:3 ratio and matching for age, controls with HIV were selected from the same study. Children with severe anemia ( < 7.0 g/dL), low body weight (weight for age z-score < −3), or chronic diseases other than TB were excluded.

#### 2.1.2 NIH Stool4TB cohort, Mbabane, Eswatini (validation cohort)

The NIH Stool4TB pediatric cohort was a prospective case-control study conducted from 2020 to 2023 in Mbabane, Eswatini (Kay et al., 2024). The study included children with and without HIV who were either clinically diagnosed or microbiologically confirmed to have TB, as well as their household contacts without TB. Microbiologically confirmed TB was defined as a positive Mtb culture or an Xpert MTB/RIF-positive result; clinical/unconfirmed TB cases were defined by the presence of symptoms, tuberculosis exposure, and chest radiograph interpretation consistent with international guidelines (Graham et al., 2015). Controls were children without HIV investigated for active TB in whom TB was ruled out according to national guidelines.

For this secondary analysis, we used residual plasma samples and clinical data from participants in the LCS and the NIH Stool4TB study after study completion. In the LCS, we included all children who developed TB during the observation period and matched controls by age. From the Stool4TB cohort, we selected participants with microbiologically confirmed and clinically/unconfirmed TB, with or without HIV, as cases and matched by age from the same household with no TB and no HIV as controls for analysis.

### 2.2 Laboratory measurement of plasma kynurenine and tryptophan

#### 2.2.1 ELISA for plasma K/T ratio

Plasma samples from the LCS were tested at the National Institute for Communicable Diseases in Johannesburg, South Africa, and NIH stoo4TB samples were tested at Baylor College of Medicine in Houston, Texas. We measured plasma kynurenine and tryptophan concentrations using a commercial ELISA kit (ImmuSmol, Inc., France). All samples were analyzed in duplicate. Per the instructions for use, acylated or derivatized plasma kynurenine and tryptophan competed with solid-phase–bound analytes for antibody binding during a 15-h incubation (overnight). Following incubation, plates were washed thoroughly to remove unbound antigen and antigen–antibody complexes. Detection was performed using an anti-rabbit IgG conjugated to horseradish peroxidase (HRP). The enzymatic reaction was initiated by adding the 3,3′,5,5′-tetramethylbenzidine (TMB) substrate and stopped after 2 min of development. Optical density (OD) was measured at 450 nm using a calibrated microplate reader. Quantification of kynurenine and tryptophan concentrations in unknown samples was achieved by interpolation from standard curves generated using calibrators provided with the assay kit. Results were expressed in μmol/L. The K/T ratio was calculated by dividing the concentration of kynurenine by that of tryptophan. During analysis, investigators were blinded to the participants’ TB status.

### 2.3 Statistical analysis

Normally distributed data are presented as the mean ± standard deviation (SD), while non-normally distributed data are shown as the median with the interquartile range (IQR). A Student’s *t*-test was used to compare the means of the variable between the two groups. Wilcoxon, Mann-Whitney and Kruskal-Wallis with Dunn’s *post-hoc* tests were used to identify significant differences between non-parametric paired and unpaired groups. Sensitivity, specificity, positive predictive value, and negative predictive value were calculated. Receiver operating characteristic (ROC) curves and Youden’s index (J) as well as PPV and NPV were used to determine the most appropriate diagnostic cut-off value for the K/T ratio to distinguish between children with active TB and those without TB.

We assessed the association between plasma K/T ratio and clinical parameters, including body mass index (BMI)-z score, CD4 count, and viral load, using Spearman’s correlation. We modelled factors associated with higher plasma K/T ratio using univariate and multivariate linear regression to estimate parameter values and standard errors (SEs). The covariates included TB cases versus controls, age, CD4 count, BMI, and viral load (log10). We used GraphPad Prism 10 (GraphPad Software, USA) and SAS Enterprise Guide 7.15 (SAS Institute, USA) for analysis.

### 2.4 Ethics

The LCS was approved by the Johns Hopkins University Institutional Review Board and the University of the Witwatersrand Human Research Ethics Committee (M10336). The NIH Stool4TB study received approval from the Eswatini Health and Human Research Review Board and the Baylor College of Medicine Biomedical Research and Assurance Information Network. Written consent was obtained from all participants or their parents/guardians. Children above the age thresholds set by the ethics boards provided written assent for participation.

## 3 Results

### 3.1 Participants’ demographics and clinical parameters

Participant demographic and clinical characteristics of both cohorts are summarized in Table 1. In the LCS group (*optimization cohort*), microbiologically confirmed, probable, and possible TB cases were combined as active TB cases. Of the 23 active TB cases, 11 (48%) were microbiologically confirmed by culture or the presence of acid-fast bacilli in sputum, while the other 12 (52%) were diagnosed clinically. Of the total, 19 (83%) had pulmonary TB, while 4 (17%) had extrapulmonary TB. All cases with TB and controls were receiving antiretroviral treatment (ARVs). No child with active TB disease had received IPT prior to enrollment in the study. Lower body mass index and CD4 cell count were evident in children with TB compared with controls.

**TABLE 1:** Subjects demographics and clinical data.

	Lung cohort study (South Africa)			NIH Stool4TB cohort (Eswatini)		
	TB cases *(n = 23)*	Controls *(n = 75)*	*P-*value	TB cases *(n = 35)*	Controls *(n = 25)*	*P-*value
Gender						
Female	16 (70%)	27 (73%)		20 (57%)	14 (56%)	
Male	7 (30%)	28 (37%)		15 (43%)	11 (44%)	
Age (years)*^A^*	10 ± 4	10 ± 4	0.6566	5 ± 8	6 ± 2	0.3567
Body Mass Index-z score (BMIz)*^B^* (kg/m^2^)	2.1 [0.5–2.8]	2.5 [1.5–3]	0.0122	0.6 [−0.1 to 1]	0.02 [−1.2 to 1]	0.1226
Classification of TB disease						
Confirmed TB	11	–		15	–	
Possible/Probable TB	12	–		20	–	
Site of TB						
Pulmonary TB	18	–		35	–	
Extrapulmonary TB	4	–		0	–	
ART regimen						
Proportion of individuals on/off ART	21/2	75/0		7/28	0/25	**0.0347**
Number of days on ART (days)*^B^*	32 (20–101)	87 (41–220)	0.0007	269 (57 to 923)	–	–
Isoniazid preventive treatment (IPT)						
Proportion of individuals on/off IPT	0/23	27/75	**< 0.0001**	3/32	0/25	0.2585
Number of days on IPT (days)*^A^*	0	63 ± 100	< **0.0001**	–	–	–
Immunological and virological markersC						
CD4 cell count (cells/mL)*^B^*	212 (211–401)	417 (300–593)	**0.0119**	–		
HIV Viral load (copies/mL)*^B^*	5368 (101–49,022)	525 (51–6,813)	**0.0148**	–		

*^A^*Mean ± SD;

*^B^*Median (interquartile range).

*^C^*Not collected in the NIH Stool4TB cohort. Bold *p*-values indicate statistically significant associations (*p* < 0.05). ART, antiretroviral therapy; IPT, isoniazid preventive therapy.

From the NIH stool4TB cohort (validation cohort), we received residual plasma samples from 60 participants including 35 (58%) with active TB, of which 15 (43%) were microbiologically confirmed by a positive sputum culture, Xpert MTB/RIF, or stool qPCR and 20 (58%) with unconfirmed TB. Controls were household contacts of the TB cases in whom TB was ruled out based on negative results from sputum Xpert Ultra, sputum culture, urine Lipoarabinomannan tests, and stool qPCR and 6-months longitudinal follow-up without the emergence of incident TB. Of the 35 active TB cases, 15 (43%) were living with HIV, and 7 (20%) were on antiretroviral therapy (ARV). All participants with HIV started ARV during the study period.

### 3.2 Plasma K/T ratio is elevated in children living with HIV who have active TB

**FIGURE 1:**
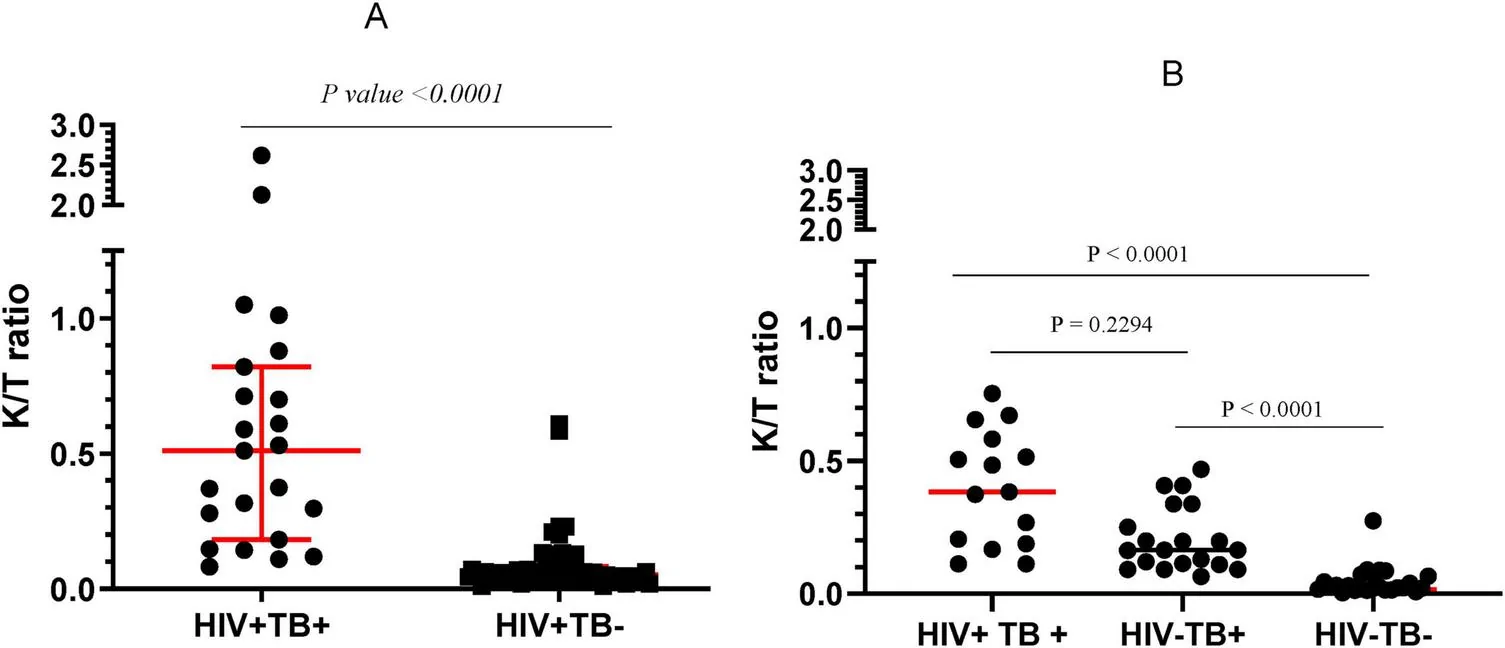
Plasma kynurenine/tryptophan (K/T) ratio measured by ELISA in plasma samples from the lung cohort study **(LCS) (A)** and the Stool4TB cohort **(B)**. **(A)** K/T ratio in children with HIV who had confirmed and unconfirmed TB (*n* = 23) compared with controls (*n* = 75) in the LCS. **(B)** Comparison of K/T ratio in children living with HIV who had confirmed TB (*n* = 15) and in those without HIV who had active TB (*n* = 20) with those who had no HIV and no TB (*n* = 25). The top and bottom red lines show the 25th and 75th percentiles, respectively, with the median shown.

In the optimization cohort, we examined whether plasma K/T ratio was increased at the time of TB diagnosis in children living with HIV with active TB. Children with HIV-associated TB had a significantly higher plasma K/T ratio (median 0.511 [IQR 0.182–0.820]) than controls with HIV and no TB (median 0.053 [IQR 0.030–0.072], *p* < 0.0001) (Figure 1A). There were no children without HIV included in this cohort.

In the validation cohort, similarly, participants with active TB, with or without HIV, had significantly higher K/T ratios than controls without HIV (Figure 1B). The median plasma K/T ratio for children with HIV-associated TB, TB without HIV, and healthy controls was 0.398 [IQR 0.188–0.581], 0.163 [IQR 0.110–0.315], and 0.024 [IQR 0.015–0.068] (*p* < 0.0001), respectively.

Combining all TB cases and controls from both cohorts, the median plasma K/T ratio for TB cases (living with or without HIV) was 0.307 [IQR 0.146–0.543] compared with 0.052 [IQR 0.031–0.072] for controls without HIV (*P* < 0.0001) (Supplementary Table 1).

Additionally, we compared plasma K/T ratio from participants with microbiologically confirmed TB with clinically diagnosed/unconfirmed TB in the optimization and validation cohorts. In the optimization cohort, microbiologically confirmed TB cases showed a trend toward higher plasma K/T ratios compared with clinically diagnosed TB cases, although the difference did not reach statistical significance (*P* = 0.0792). In the validation cohort, microbiologically confirmed TB cases had significantly higher K/T ratios than clinically diagnosed TB cases (*P* = 0.0290; Supplementary Figure 1).

### 3.3 Diagnostic value of plasma K/T ratio in children living with HIV in the optimization cohort

**FIGURE 2:**
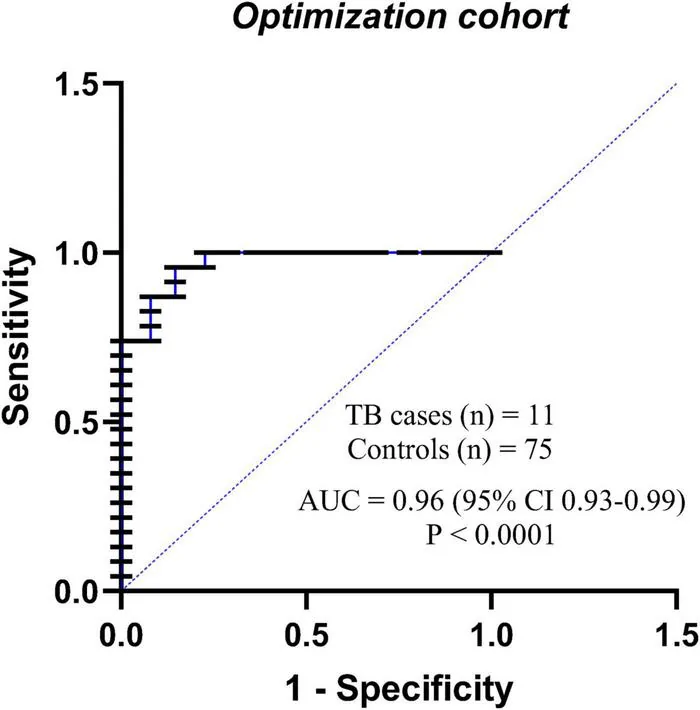
Receiver operating characteristic (ROC) curve of plasma K/T ratio for detecting confirmed TB disease among children living with HIV in the lung cohort study. Applying a cut-off value of 0.100, plasma K/T ratio gave a sensitivity of 95% (CI 79–99), specificity of 85% (CI 0.75–0.91), PPV of 85%, NPV of 98%, and AUC of 0.96 (95% CI 0.93–0.99).

Next, we evaluated the diagnostic utility of an elevated plasma K/T ratio for detecting active TB disease in children with HIV, using only data from the LCS. Using data from participants with microbiologically confirmed TB only (cases) and data from participants without TB (controls), we calculated sensitivity, specificity, Youden’s index (J), positive predictive value (PPV), and negative predictive value (NPV) for identifying active TB (Table 2). A ROC curve was used to determine the optimal cut-off for TB diagnosis. At a cut-off of 0.100, the K/T ratio achieved an optimal sensitivity of 95% (CI 0.79–0.99), specificity of 85% (CI 0.75–0.91), PPV of 85%, and NPV of 98% for diagnosing active TB. The ROC curve analysis resulted in an AUC of 0.96 (CI 0.93–0.99, *P*-value < 0.0001), as shown in Figure 2.

**TABLE 2:** Diagnostic significance of K/T ratio for active TB case detection in children in lung cohort study.

		Plasma K/T ratio for diagnosing active TB disease						
Cut-off	Sensitivity (%)	95% CI	Specificity (%)	95% CI	Youden’s index (J)	95% CI	PPV (%)	NPV (%)
0.010	100	0.85–1.00	2.7	0.00–0.09	0.03	0.00–0.09	20	100
0.020	100	0.85–1.00	10	0.05–0.10	0.10	0.03–0.17	24	100
0.030	100	0.85–1.00	22	0.14–0.33	0.22	0.12–0.32	31	100
0.040	100	0.85–1.00	33	0.23–0.44	0.33	0.22–0.44	32	100
0,050	100	0.85–0.99	45	0.34–0.56	0.45	0.33–0.57	37	100
0,060	100	0.85–0.99	62	0.57–0.66	0.62	0.51–0.73	48	99
0,070	100	0.85–0.99	77	0.66–0.79	0.77	0.68–0.86	60	99
0,080	98	0.88–0.98	84	0.75–0.85	0.82	0.73–0.91	76	99
**0,090**	**97**	**0.82–0.98**	**95**	**0.78–0.85**	**0.92**	**0.84–1.00**	**82**	**98**
**0,100**	**95**	**0.79–0.97**	**85**	**0.75–0.91**	**0.80**	**0.69–0.91**	**85**	**98**
0.115	90	0.75–0.96	85	0.75–0.91	0.75	0.62–0.88	86	98
0,125	86	0.67–0.95	85	0.75–0.90	0.71	0.58–0.84	87	97
0,150	82	0.62–0.83	92	0.83–0.92	0.74	0.62–0.86	87	97
0,200	73	0.53–0.87	93	0.85–0.93	0.66	0.52–0.80	91	96
0,225	80	0.53–0.85	95	0.90–0.95	0.75	0.63–0.87	92	96
0,250	72	0.50–0.81	96	0.95–0.99	0.68	0.54–0.82	94	95
0,300	65	0.44–0.80	100	0.95–0.99	0.65	0.50–0.80	94	95

TB positives: children with lab confirmed active TB (*n* = 11) at time of TB diagnosis. Children with no TB: controls no TB (*n* = 75). Bold values indicate the selected K/T ratio cut-off and corresponding diagnostic performance estimates.

### 3.4 Diagnostic value of plasma K/T ratio in children living with or without HIV in the validation cohort

**FIGURE 3:**
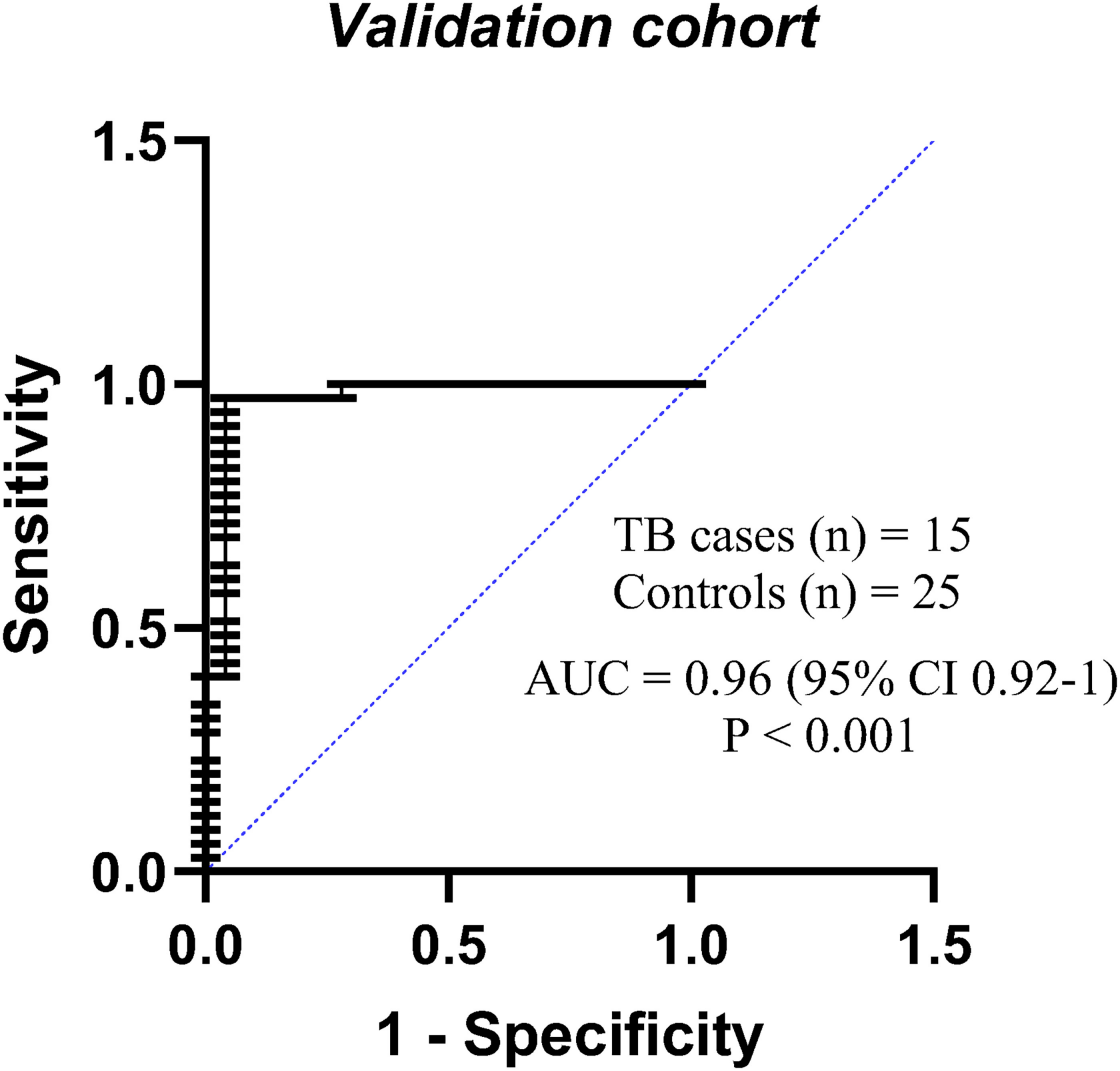
Receiver operating characteristic curve (ROC) of plasma K/T ratio for detecting the presence of confirmed active TB disease in the validation cohort. Using a cut-off value of 0.100, plasma K/T ratio gave a sensitivity of 95% (CI 0.79–0.99), specificity of 85% (CI 0.75–0.91), PPV of 85%, and NPV of 98% with an AUC of 0.9691 (95% CI 0.92–1.00).

We assessed the diagnostic performance of plasma K/T ratio (above the predefined threshold) for detecting active TB in the NIH stool4TB validation cohort. We combined results for participants with and without HIV who had microbiologically confirmed active TB only (cases) and results for participants with no TB and no HIV (controls). We calculated sensitivity, specificity, Youden’s index (J), PPV, and NPV to identify active TB (Table 3). A ROC curve to determine the optimal cut-off point for TB diagnosis yielded an AUC of 0.9691 (*P* < 0.0001; 95% CI 0.92–1.00) (Figure 3).

**TABLE 3:** Diagnostic performance of the plasma K/T ratio for detecting microbiologically confirmed TB among children with HIV in the validation cohort.

		Plasma K/T ratio for diagnosing active TB disease						
Cut-off	Sensitivity (%)	95% CI	Specificity (%)	95% CI	Youden’s index (J)	95% CI	PPV (%)	NPV (%)
0.010	100	0.85–1.00	2.7	0.00–0.09	0.03	0.00–0.09	20	100
0.020	100	0.85–1.00	10	0.05–0.10	0.10	0.03–0.17	24	100
0.030	100	0.85–1.00	22	0.14–0.33	0.22	0.12–0.32	31	100
0.040	100	0.85–1.00	33	0.23–0.44	0.33	0.22–0.44	32	100
0,050	100	0.85–0.99	45	0.34–0.56	0.45	0.33–0.57	37	100
0,060	100	0.85–0.99	62	0.57–0.66	0.62	0.51–0.73	48	99
0,070	100	0.85–0.99	77	0.66–0.79	0.77	0.68–0.86	60	99
0,080	98	0.88–0.98	84	0.75–0.85	0.82	0.73–0.91	76	99
**0,090**	**97**	**0.82–0.98**	**95**	**0.78–0.85**	**0.92**	**0.84–1.00**	**82**	**98**
**0,100**	**95**	**0.79–0.97**	**85**	**0.75–0.91**	**0.80**	**0.69–0.91**	**85**	**98**
0.115	90	0.75–0.96	85	0.75–0.91	0.75	0.62–0.88	86	98
0,125	86	0.67–0.95	85	0.75–0.90	0.71	0.58–0.84	87	97
0,150	82	0.62–0.83	92	0.83–0.92	0.74	0.62–0.86	87	97
0,200	73	0.53–0.87	93	0.85–0.93	0.66	0.52–0.80	91	96
0,225	80	0.53–0.85	95	0.90–0.95	0.75	0.63–0.87	92	96
0,250	72	0.50–0.81	96	0.95–0.99	0.68	0.54–0.82	94	95
0,300	65	0.44–0.80	100	0.95–0.99	0.65	0.50–0.80	94	95

TB positives: children with lab confirmed active TB (*n* = 11) at time of TB diagnosis. Children with no TB: controls no TB (*n* = 75). Bold values indicate the selected K/T ratio cut-off and corresponding diagnostic performance estimates.

### 3.5 Diagnostic value of plasma K/T ratio in children without HIV in the validation cohort

**FIGURE 4:**
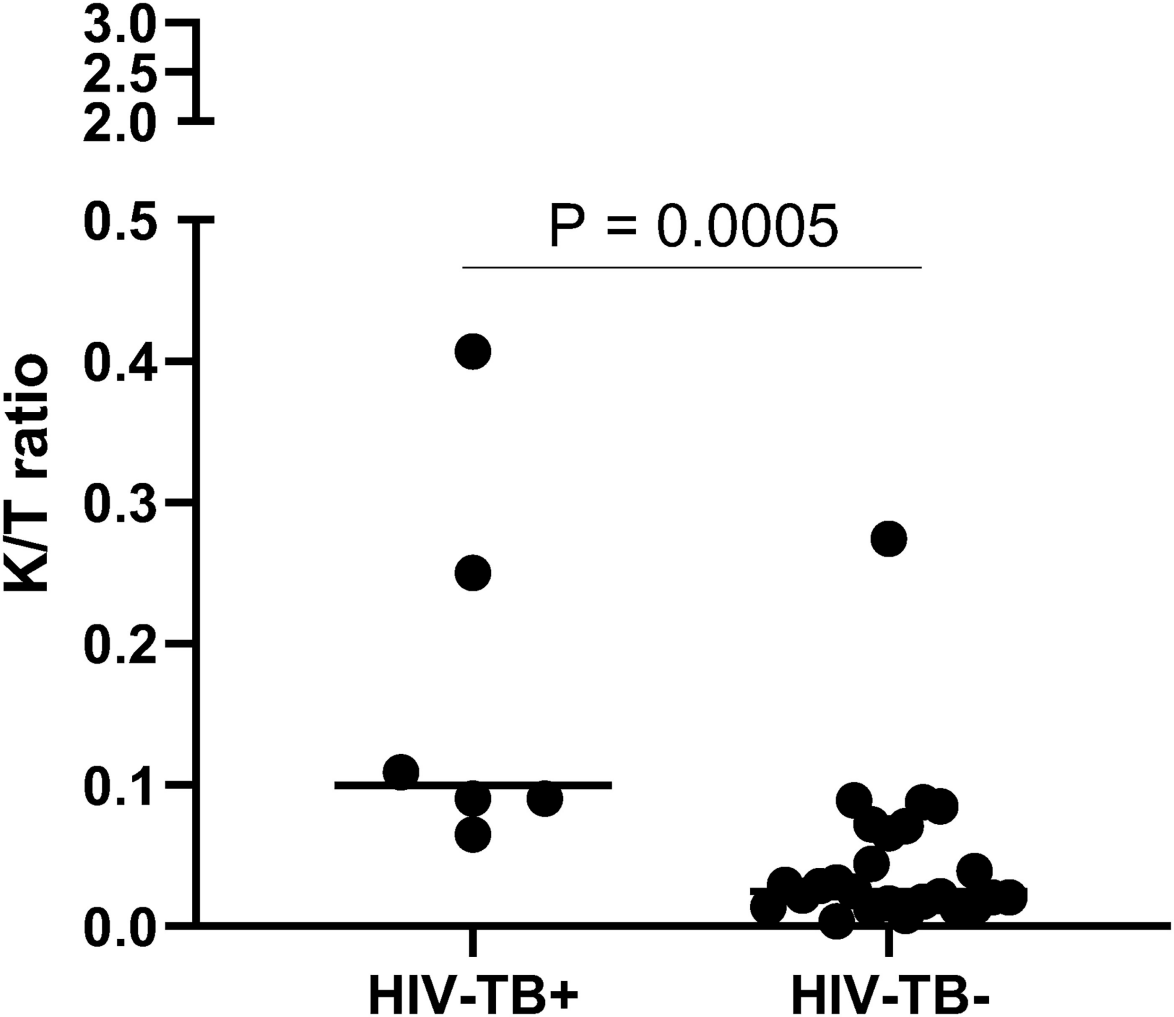
Plasma kynurenine/tryptophan (K/T) ratio measured by ELISA in plasma samples from the validation cohort. K/T ratio in children without HIV who had confirmed TB (*n* = 6) compared with those without HIV or TB (*n* = 25). Children with TB had significantly higher plasma K/T ratio compared to controls, *P* = 0.0005. The top and bottom red lines show the 25th and 75th percentiles, respectively, with the median shown.

**FIGURE 5:**
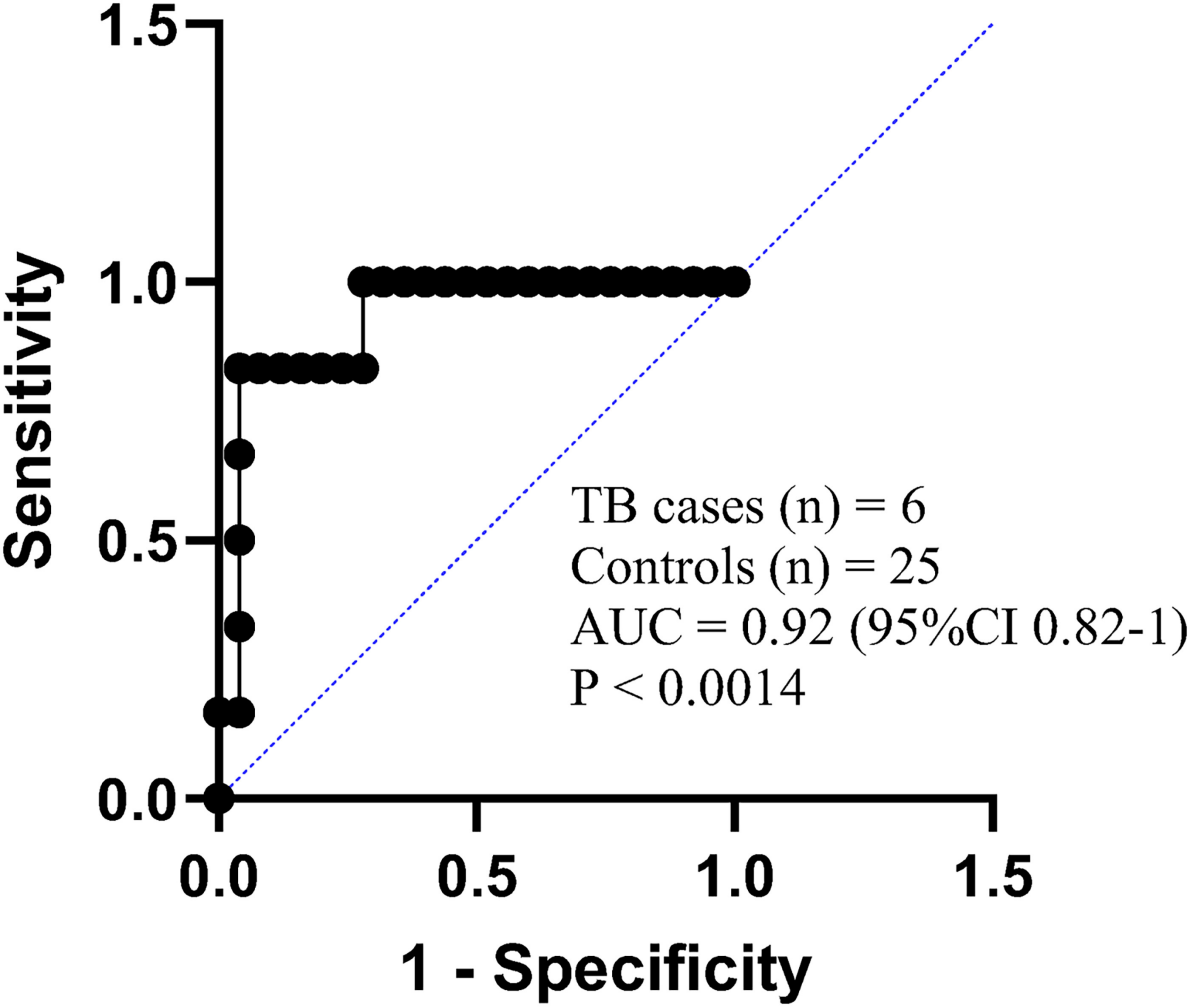
Receiver operating characteristic (ROC) curve of plasma K/T ratio for detecting confirmed TB disease among children without HIV in the validation cohort. Applying a cut-off value of 0.090, plasma K/T ratio gave a sensitivity of 83 % (CI 0.43–0.99), specificity of 96% (CI 0.80–0.99), PPV of 96%, NPV of 90%, and AUC of 0.92 (95% CI 0.82–1) for laboratory confirmed TB.

Next, we evaluated the diagnostic performance of the plasma K/T ratio for detecting active TB among children without HIV in the validation cohort using only microbiologically confirmed TB cases. Diagnostic performance was assessed by calculating sensitivity, specificity, positive predictive value (PPV), negative predictive value (NPV), and Youden’s index across a range of K/T ratio cut-offs (Table 4). HIV-negative children with microbiologically confirmed TB had significantly higher plasma K/T ratios (median 0.099 [IQR 0.084–0.289]) than HIV-negative controls without TB (median 0.024 [IQR 0.015–0.068] *P* = 0.0005) (Figure 4). A ROC curve analysis demonstrated excellent discriminatory performance, with an area under the curve (AUC) of 0.92 (95% CI: 0.82–1.00; *P* = 0.0014) (Figure 5). Using a K/T ratio cut-off of 0.090 yielded the highest Youden Index in the HIV-negative subgroup (*J* = 0.79) with a sensitivity of 83% (0.43–0.99), specificity of 96% (0.80–0.99), PPV of 96% and NPV of 90%, Table 5.

**TABLE 4:** Diagnostic significance of plasma K/T ratio in diagnosing active TB in the validation cohort. TB positives: all microbiologically confirmed active TB (*n* = 15). TB negatives: controls (no TB, no HIV) (*n* = 25).

Cut-off	Sensitivity (%)	95% CI	Specificity (%)	95% CI	Youden’s index (J)	95% CI	PPV (%)	NPV (%)
0.010	100	0.90–1.00	8	0.04–0.20	0.08	0.00–0.19	18	100
0.020	100	0.90–1.00	26	0.20–0.45	0.26	0.09–0.43	20	100
0.030	100	0.90–1.00	60	0.40–0.76	0.60	0.41–0.79	27	100
0.040	100	0.90–1.00	68	0.48–0.80	0.68	0.50–0.86	30	100
0,050	100	0.90–099	72	0.52–0.85	0.72	0.55–0.89	35	100
0,060	97	0.85–0.99	72	0.52–0.84	0.69	0.50–0.88	47	97
0,070	97	0.85–0.99	78	0.60–0.86	0.75	0.58–0.92	51	96
0,080	96	0.84–0.98	84	0.70–0.88	0.80	0.65–0.95	56	96
**0,090**	**94**	**0.81–0.98**	**90**	**0.80–0.95**	**0.84**	**0.69–0.99**	**78**	**94**
**0,100**	**88**	**0.74–0.95**	**96**	**0.90–0.97**	**0.84**	**0.66–1.00**	**82**	**94**
0,125	78	0.57–0.85	96	0.90–0.98	0.74	0.55–0.93	84	91
0,150	74	0.54–0.83	95	0.80–0.98	0.69	0.49–0.89	87	90
0,200	58	0.33–0.63	96	0.80–0.98	0.54	0.30–0.78	93	90
0,225	52	0.27–0.60	96	0.80–0.99	0.48	0.23–0.73	93	90
0,250	44	0.25–0.56	96	0.82–0.99	0.40	0.14–0.66	95	89
0,300	40	0.25–0.54	100	0.86–1.00	0.40	0.15–0.65	96	78

Bold values indicate the selected K/T ratio cut-off and corresponding diagnostic performance estimates.

**TABLE 5:** Diagnostic performance of the plasma K/T ratio for detecting microbiologically confirmed TB among HIV-negative children in the validation cohort.

Cut-off	Sensitivity (%)	95% CI	Specificity (%)	95% CI	Youden’s index (J)	95% CI	PPV (%)	NPV (%)
0.010	100	0.60–1.00	8	0.01–0.24	0.08	0.02–0.30	100	10
0.020	100	0.60–1.00	36	0.20–0.55	0.36	0.20–0.52	100	20
0.030	100	0.60–1.00	60	0.40–0.76	0.60	0.44–0.76	100	30
0.040	100	0.60–1.00	68	0.48–0.82	0.68	0.52–0.84	100	40
0.050	100	0.60–1.00	72	0.52–0.85	0.72	0.56–0.88	100	50
0.060	83	0.43–0.99	72	0.52–0.85	0.55	0.56–0.88	96	60
0.070	83	0.43–0.99	80	0.60–0.91	0.63	0.32–0.95	96	70
0.080	83	0.43–0.99	84	0.65–0.93	0.67	0.36–0.99	96	80
**0.090**	**83**	**0.43–0.99**	**96**	**0.80–0.99**	**0.79**	**0.45–1.00**	**96**	**90**
**0.100**	**67**	**0.30–0.94**	**96**	**0.80–0.99**	**0.62**	**0.41–0.96**	**92**	**100**
0.120	50	0.18–0.81	96	0.80–0.99	0.46	0.29–0.96	89	100
0.150	33	0.059–0.70	96	0.80–0.99	0.29	0.14–0.78	86	100
0.175	16	0.008–0.56	96	0.80–0.99	0.12	0.00–0.60	83	100
0.200	16	0.008–0.56	100	0.86–1.00	0.16	0.00–0.39	83	100
0.300	6	0.004–0.23	100	0.86–1.00	0.16	0.00–0.46	83	100

TB positives: children with microbiologically confirmed active TB (*n* = 6). Children with no HIV and no TB: controls (*n* = 25). Bold values indicate the selected K/T ratio cut-off and corresponding diagnostic performance estimates.

### 3.6 Association of plasma K/T ratio with age, body mass index, CD4 count, and HIV viral load

We examined the association of the plasma K/T ratio with BMI-z score, CD4 count, and HIV viral load at the time of TB diagnosis using samples from the LCS. In Spearman’s correlation analysis among patients diagnosed with active TB and controls, we found no correlation between the K/T ratio and age, BMI-z score, CD4, or HIV viral load. This analysis was not performed in the NIH stool4TB validation cohort because limited CD4 and viral load data were not available for all participants. For age and BMI-z score, there was no significant correlation.

### 3.7 The effect of isoniazid prophylaxis on K/T ratio in children with HIV

**FIGURE 6:**
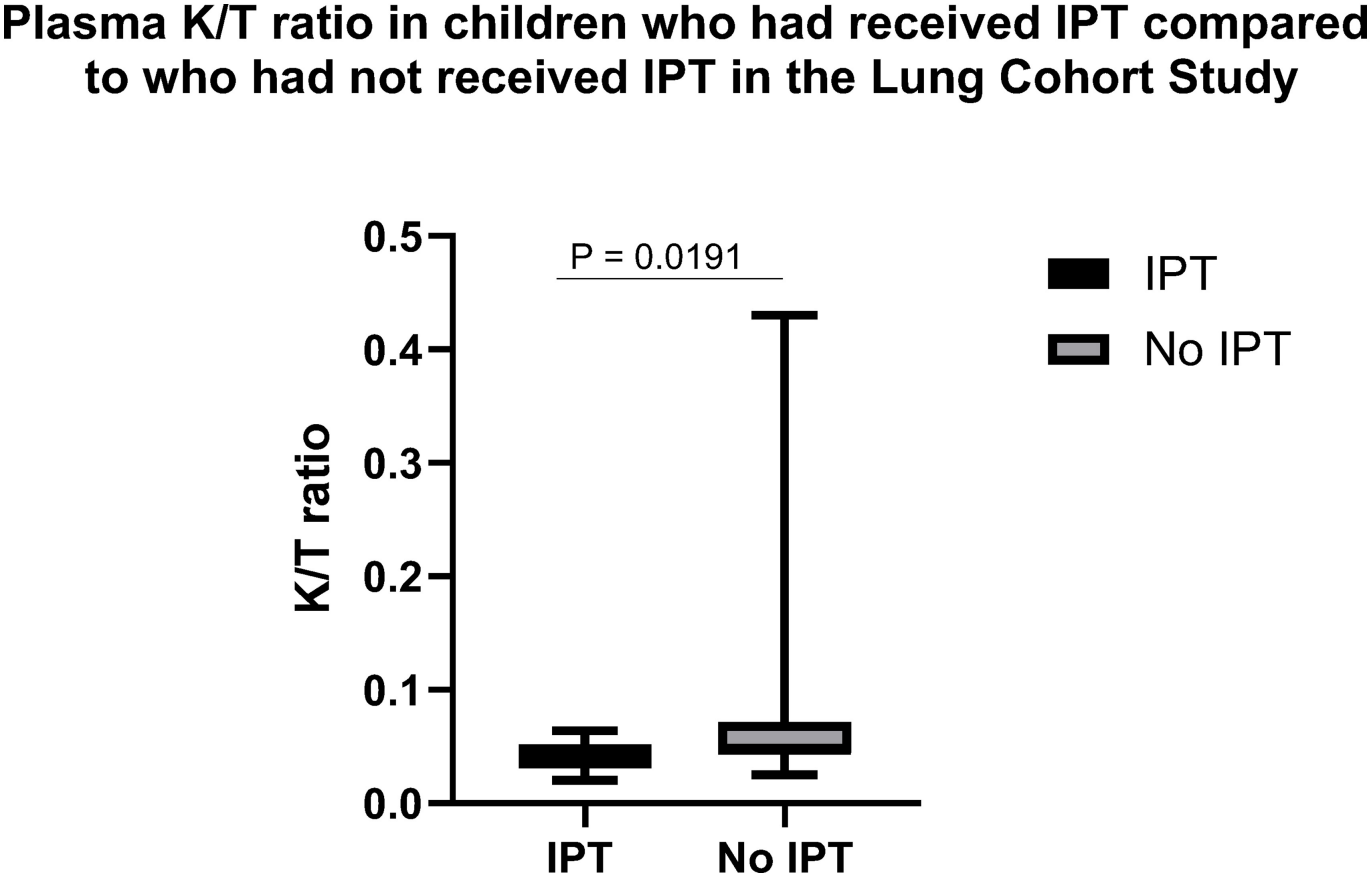
Comparison of plasma K/T ratio in children who had received Isoniazid preventative therapy (IPT) (*n* = 27) to those who did not (*n* = 48) in the LCS. The top and bottom lines of the boxes show the 25th and 75th percentiles, respectively, with the median shown.

Among participants in the LCS, no child diagnosed with active TB had received IPT; however, among the controls, 25% (27/75) had received IPT. Despite the small sample size, there was a significant difference in plasma K/T ratios between controls who received IPT and those who did not. The median plasma K/T ratio for controls on IPT was 0.041 [IQR 0.031–0.052], compared to 0.047 [0.045–0.071], (*p* = 0.0191, Figure 6).

### 3.8 The effect of ART on K/T ratio in children with HIV

Next, using data from participants in the LCS, we examined whether being on ART and the duration of ART influenced the plasma K/T ratio in children with active TB at diagnosis. Only two children with active TB were not on ART at diagnosis. These two had higher K/T ratios of 2.150 and 1.011. We found no significant difference in the number of days on ART between participants with active TB and controls (*P* = 0.8214). Additionally, we examined whether there was a correlation between plasma K/T ratio and number of days on ART. We found no significant correlation (*P* = 0.4146) between plasma K/T ratio and number of days on ART.

### 3.9 Predictors of elevated plasma K/T ratio in both cohorts

To determine whether the association between plasma K/T ratio and active TB was independent of other clinical factors, a multivariable logistic regression model was fitted including age, BMI, CD4 count, and viral load.

In univariate analysis, BMI and TB status were significantly associated with plasma K/T ratio (Table 4). Children from the validation cohort had higher K/T ratios compared with those from the optimization cohort (β = 0.068, *p* = 0.0218), while children with TB demonstrated substantially elevated plasma K/T ratios compared those with no TB (β = 0.228, *p* < 0.0001) (Table 4). Higher BMI was associated with lower K/T ratio (β = −0.010, *p* = 0.0124). After multivariable adjustment, three factors remained independently associated with higher plasma K/T ratio (Table 6). Children from the validation cohort had significantly higher K/T ratios than those from the LCS cohort (β = 0.137, *p* = 0.0045). HIV-positive status was independently associated with increased plasma K/T ratio (β = 0.176, *p* < 0.0001), and TB-positive status remained the strongest predictor of elevated K/T ratio (β = 0.190, *p* < 0.0001). Age, BMI, CD4 count, and viral load were not independently associated with K/T ratio after adjustment. The final model explained 44.1% of the variance in plasma K/T ratio (R^2^ = 0.441). Diagnostic assessment demonstrated no evidence of problematic multicollinearity, with variance inflation factor, tolerance, and conditional index values all within acceptable limits. ART and IPT exposure could not be included in the multivariable model because treatment data were incomplete for a subset of participants.

**TABLE 6:** Independent predictors of elevated plasma K/T ratio in both the optimization and validation cohorts.

Variable	Univariate		Multivariate	
	β(SE)	*P*-Value	β(SE)	*P*-Value
**Age**	−0.004 (0.003)	0.2250	–	–
Group				
Optimization cohort	Ref	–	Ref	–
Validation cohort	0.068 (0.029)	**0.0218**	0.137 (0.048)	**0.0045**
HIV status				
Negative	Ref	–	Ref	–
Positive	0.050 (0.032)	0.1235	0.176 (0.044)	**< 0.0001**
BMI Class				
Underweight	0.055 (0.030)	0.0671	–	–
Normal	Ref	–	–	–
Overweight	−0.063 (0.069)	0.3590	–	–
**BMI**	−0.010 (0.004)	**0.0124**	−0.00002 (0.004)	0.9956
TB status				
TB Negative	Ref	–	Ref	–
TB Positive	0.228 (0.024)	**< 0.0001**	0.190 (0.026)	**< 0.0001**
HIV severity indicators				
CD4 count	−0.00002 (0.00008)	0.7716	–	–
Log10 Viral load	−0.024 (0.015)	0.1003	–	–

R-Square = 0.441; Model diagnostics of VIF (variance inflation factor), tolerance, and conditional index are satisfied. Bold *p*-values indicate statistically significant associations (*p* < 0.05).

### 3.10 Reproducibility of K/T ratio ELISA across laboratories

We evaluated intra-run and inter-laboratory reproducibility of the ELISA assay in the NICD and Houston laboratories using kit-supplied control samples. For intra-run reproducibility, samples were tested in duplicate within the same run over five consecutive days, and assay variability was assessed using standard deviation (SD) and coefficient of variation (CV). Intra-run CVs ranged from 1.5–4.2% for tryptophan and 0.8–5.3% for kynurenine, indicating good assay precision. Inter-laboratory precision testing similarly demonstrated good reproducibility of kynurenine, tryptophan, and K/T ratio measurements (Supplementary Table 1). For Kynurenine, the inter-run %CV for low- and high-level control samples were 4.7% and 5.7%, respectively. Tryptophan also exhibited inter-run % CVs of 6.1% and 3.2%, while K/T ratios showed inter-run CVs of 5.2% and 5.1% for the low- and high-level controls, respectively. Collectively, these findings demonstrate good assay precision and reproducibility across laboratories, supporting the reliability of biomarker measurements used in subsequent analyses. Standardized calibration and quality-control procedures were maintained throughout testing.

## 4 Discussion

Non-sputum-based tests are crucial for diagnosing and predicting active TB in children. In this study, we evaluated plasma K/T ratio as a tool for detecting active TB in children with and without HIV in two distinct cohorts. At the time of TB diagnosis, plasma K/T ratio was significantly higher in children with active TB compared to controls in both the optimization and validation cohorts. An elevated plasma K/T ratio in children with HIV-associated TB is consistent with similar findings in other populations (Adu-Gyamfi et al., 2017, 2020, 2021; Almeida et al., 2009; Gautam et al., 2018; Tornheim et al., 2022). Our team and others have shown that increased IDO activity, measured as the product-to-substrate (K/T ratio), serves as a promising diagnostic biomarker for active TB in both HIV-infected and uninfected adults (Adu-Gyamfi et al., 2017, 2020, 2021; Suzuki et al., 2013).

A key finding of this study is the reproducible identification of a plasma K/T ratio cut-off of 0.100 as optimal for distinguishing active TB from controls across independent cohorts comprising children living with HIV. For children without HIV, the Youden’s index identified a threshold of 0.090 as providing the best overall balance between sensitivity and specificity however the difference in performance between the 0.090 and 0.100 cut-offs was minimal.

As expected, the highest discriminatory performance was observed in the optimization cohort, which included a smaller, more homogeneous group of microbiologically confirmed cases all living with HIV, whereas in the validation cohort, discriminatory performance was improved by using the cutoff of 0.10 when all children with confirmed TB were included but a lower cutoff of 0.090 when only children without HIV were included. Collectively, these results suggest that the K/T ratio is a promising biomarker for TB diagnosis in children, with a threshold of 0.09–0.10 that balances sensitivity and specificity across varying clinical contexts while maintaining a favorable positive predictive value. Further validation in larger, prospectively recruited cohorts and in populations with comorbidities will be important to confirm its generalizability and clinical utility.

In both cohorts, using cut-offs of 0.090–0.100, more than 91% of children with HIV-associated TB could be distinguished from participants without active TB. Thus, the plasma K/T ratio could potentially serve as a first-line rule-out screening test for active TB in children with HIV and symptoms suggestive of TB (i.e., presumed TB cases). In adult populations, we have proposed a cut-off ranging from 0.040, for participants without HIV, to 0.080, for people with HIV (Adu-Gyamfi et al., 2020). In pregnant women with HIV, we found a higher cut-off of 0.100 more appropriate (Adu-Gyamfi et al., 2021) to diagnose active TB. Several other studies have recommended variable cut-offs in different populations, using various assays, including high-performance liquid chromatography–mass spectrometry (Adu-Gyamfi et al., 2017; Gatechompol et al., 2024; Suzuki et al., 2013).

In the subgroup of HIV-negative children, the plasma K/T ratio demonstrated excellent diagnostic performance for distinguishing microbiologically confirmed TB from controls. These findings complement our multivariable analyses, in which TB disease remained the strongest independent predictor of an elevated K/T ratio after adjustment for potential confounders, supporting the hypothesis that TB itself is the principal driver of kynurenine pathway activation. Although sensitivity at the selected cut-off (0.090) was lower than that observed in the overall validation cohort, specificity (96%) and the negative predictive value (90%) remained high, indicating potential utility for excluding active TB in HIV-negative children. Nevertheless, the subgroup included only six microbiologically confirmed TB cases, limiting the precision of the diagnostic estimates. Prospective studies with larger HIV-stratified cohorts are warranted to confirm these findings and establish whether HIV status influences the optimal diagnostic threshold or clinical performance of the K/T ratio. Although HIV-stratified analyses were performed, the small number of microbiologically confirmed HIV-negative TB cases limited the precision of the diagnostic accuracy estimates. Consequently, these subgroup findings should be considered exploratory and require validation in larger independent cohorts.

The WHO has established specific Target Product Profiles (TPPs) and recommendations for TB diagnostic tests in children, emphasizing the need for non-sputum-based tests to overcome the paucibacillary nature of pediatric TB. For the detection of active TB in children using a non-sputum-based specimen, the WHO recommends a minimum 80% (low complexity) to 65% (point-of-care) sensitivity. For screening tests to rule out TB or determine who needs further confirmatory testing, the WHO recommends a minimum sensitivity of 90% and specificity of 70%. Hence, plasma K/T ratio is a promising candidate biomarker for a non-sputum based triage test in children completing evaluation for active TB. In children with HIV, the plasma K/T ratio affords acceptable accuracy for the diagnosis of TB, meeting the WHO TPP specifications by achieving superior sensitivity and specificity compared with existing commercial diagnostic tools (World Health Organization [WHO], 2014). In children without HIV, results are promising but require further exploration in additional studies.

In this study, we made several observations with clinical relevance. First, we observed no correlation between plasma K/T ratio and other clinical parameters, including BMI-z score, CD4, and HIV viral load, in either controls or cases, unlike adult studies that reported a correlation between plasma K/T ratio and BMI (Adu-Gyamfi et al., 2020, 2021). This suggests plasma K/T ratio could provide robust results in children regardless of nutritional or HIV status. These findings indicate that the association between plasma K/T ratio and TB disease is independent of the clinical variables evaluated in this study, suggesting that the biomarker may provide additional information beyond these factors.

Multivariable analysis demonstrated that TB disease, HIV infection, and cohort site were independently associated with higher plasma K/T ratio. Among these predictors, TB-positive status exhibited the largest effect size, reinforcing the central role of TB-associated immune activation and downstream disruption of nicotinamide biosynthesis pathways, which may, in part, reflect the effects of *M. tuberculosis*-derived virulence factors on host cellular metabolism (Ferrario et al., 2024). This finding is biologically plausible, as *M tuberculosis* infection stimulates interferon-γ–mediated activation of IDO-1, resulting in accelerated conversion of tryptophan to kynurenine and subsequent elevation of the K/T ratio (Almeida et al., 2009; Munn and Mellor, 2013). Previous studies have similarly reported increased K/T ratios among individuals with active TB and demonstrated declines following successful anti-TB treatment, supporting its utility as a marker of disease-associated immune activation (Almeida et al., 2009; Gatechompol et al., 2024).

Importantly, TB status remained a significant predictor of elevated K/T ratio even after adjustment for HIV infection and other potential confounders. This observation suggests that the association between K/T ratio and TB is not solely attributable to HIV-related chronic immune activation. Rather, it indicates that active TB contributes independently to perturbations in tryptophan metabolism (Suzuki et al., 2012; Tornheim et al., 2022). This finding strengthens the biological rationale for the K/T ratio as a TB-specific host-response biomarker and supports its potential diagnostic utility in populations with high HIV prevalence, where distinguishing TB-associated inflammation from HIV-associated immune activation remains a major challenge. Similar observations have been reported in studies of both HIV-infected and HIV-uninfected populations, where elevated K/T ratios were associated with active TB disease and demonstrated promising diagnostic performance (Adu-Gyamfi et al., 2017; Almeida et al., 2009; Gatechompol et al., 2024; Tornheim et al., 2022).

Interestingly, children from the validation cohort exhibited significantly higher plasma K/T ratios than those from the optimization cohort. This difference may partly reflect the higher proportion of TB cases in the validation cohort, given the strong association between TB and increased K/T ratio observed in the present study and as previously published (Adu-Gyamfi et al., 2017; Suzuki et al., 2012). Other factors known to influence kynurenine pathway activity, including nutritional status, environmental exposures, pathogen burden, host genetic factors, and background levels of immune activation, may also have contributed to the observed differences between cohorts (Badawy, 2017; Chen and Guillemin, 2009). As these variables were not systematically assessed, further studies in larger prospective cohorts are required to elucidate the factors underlying these cohort-specific differences.

Although limited to a single cohort, children in the control group who had received IPT had lower plasma K/T ratio than who had not received IPT. Similar observations have been reported among adults receiving IPT as part of routine care (Adu-Gyamfi et al., 2020, 2021). While this finding raises the possibility that INH may influence kynurenine pathway metabolism, the underlying mechanism remains uncertain. A reduction in plasma K/T ratio through Isoniazid treatment may correlate with the mechanism by which Isoniazid reduces susceptibility of active TB disease, although a direct drug side effect unrelated to TB susceptibility cannot be excluded (Huang et al., 2020). Isoniazid was originally developed as a nicotinamide analogue, and we have previously postulated a causative role of the nicotinamide synthesis pathway in TB (Suchard et al., 2020). Speculatively, administration of isoniazid might bypass the body’s need to synthesize nicotinamide through the IDO-catalyzed pathway (Suchard and Savulescu, 2022). However, because this analysis was cross-sectional and no TB cases received IPT, residual confounding cannot be excluded, and a mechanistic link between IPT, modulation of the K/T ratio, and protection against TB should be considered hypothesis-generating. Prospective longitudinal studies of children initiating IPT will be required to determine whether INH independently alters K/T ratio and whether such changes area associated with reduced TB risk.

Recent advances in pathogen-directed diagnostics, including nanopore and targeted next-generation sequencing, have shown promise for improving detection and characterization of Mtb, however, these approaches require detection of mycobacterial nucleic acids and may be limited by the paucibacillary nature of pediatric TB (Carandang et al., 2025; Notarte et al., 2026). Host-response biomarkers, such as the K/T ratio, may therefore complement molecular methods by providing information on disease-associated immune activation, supporting future multimodal diagnostic strategies.

This study has several strengths. We measured the K/T ratio in plasma, an easily collected clinical sample from children, compared with sputum. Both the optimization and validation cohorts were from a TB-endemic area; therefore, many of our controls were likely latently infected with *M tuberculosis*, although interferon-gamma release assays were not performed. Nevertheless, the K/T ratio strongly distinguished between TB patients and healthy controls, in other words, latent TB which may have been undetected in controls did not affect the utility of the K/T ratio to detect active TB. Most blood-based diagnostics such as IGRAs and tuberculin skin tests fail to differentiate individuals with active TB from those with latent *M tuberculosis* (Mehtani et al., 2021). The K/T ratio performs well in individuals with HIV, whereas most blood-based assays perform poorly in people living with HIV (Adu-Gyamfi et al., 2020, 2021; Esmail et al., 2025). The ELISA method requires no complicated equipment and is simple to perform, and could be transformed to an automated immunoassay; a limitation of the assay is that its current format requires an overnight incubation step. An important limitation of our study is that the control groups were from household contacts of TB patients and may not be representative of children presenting to hospital with other illnesses. Further work in hospital-based patient groups is recommended.

Additionally, although the K/T ratio showed promising diagnostic performance, its clinical utility may vary across settings because predictive values are influenced by TB prevalence. Furthermore, our study did not directly compare the K/T ratio with established diagnostics such as Xpert Ultra, stool PCR, urine LAM, or sequencing-based assays. Therefore, the K/T ratio should be viewed as a complementary host-response biomarker requiring further validation. As our study was conducted in high TB-burden settings, additional studies are needed to assess its performance in lower-incidence regions and among children with other infectious or inflammatory conditions that may also influence tryptophan metabolism. Also, a limitation of the cohort is that latent TB infection status was not assessed, as IGRA testing was not performed. Given evidence that immune activation and tryptophan metabolism may be altered in LTBI, inclusion of children with LTBI among controls could have influenced K/T ratio measurements and affected estimates of diagnostic accuracy. Furthermore, different exclusion criteria were applied only to controls, this may have resulted in a healthier control group and could potentially have inflated the observed diagnostic performance of the plasma K/T ratio.

These findings, together with previous work, suggest that the plasma K/T ratio is highly sensitive and can be used to screen for active TB disease in people of all ages particularly, those with HIV. Confirmation would require microbiological samples but negative results would confidently exclude TB. The presence of active TB causes a marked elevation in the K/T ratio, with even higher levels in individuals with TB-HIV co-infection. Among those who tested negative for K/T ratio (below a cut-off of 0.100), 93% did not have TB. This high NPV suggests that the K/T ratio has potential for use as a screening test in children, especially those with HIV and symptoms suggestive of TB prompting evaluation (i.e., presumed TB). The PPV of 84% suggests that the K/T ratio could help identify a subset of children in whom TB can be confirmed by a second test. A negative result, however, would reliably rule out TB in a screening triage scenario. A quantitative tool may have particular utility in longitudinal monitoring of children diagnosed with TB, an aspect warranting further study. These diagnostic strengths, combined with the use of plasma—which eliminates the need for sputum collection, a process that is often difficult in children—makes the K/T ratio a highly compelling biomarker candidate for TB screening in children, both with and without HIV.

In conclusion, our findings suggest that the plasma K/T ratio is a sensitive tool for diagnosing TB in children with HIV. Overall, the high NPV, strong PPV, and the practicality of a plasma-based test that avoids sputum collection underscore the K/T ratio’s promise as an effective screening and triage biomarker for TB diagnosis in children, including those without HIV.

## Data Availability

The raw data supporting the conclusions of this article will be made available by the authors, without undue reservation.
