# Supplementary material for: Plasma kynurenine/tryptophan ratio as a sensitive blood-based diagnostic tool for tuberculosis: a diagnostic accuracy study among children living with HIV
Source: Front Microbiol. 2026 Aug 25;17:1898324. doi: 10.3389/fmicb.2026.1898324 (PMC13550139; doi:10.3389/fmicb.2026.1898324)
Supplement: Supplementary file 1 [file Data_Sheet_1.docx]

**Supplemental tables**

|  | **Supplemental Table 1: Diagnostic significance of K/T ratio in diagnosing active TB in both cohorts** | | | | | | | |
| --- | --- | --- | --- | --- | --- | --- | --- | --- |
|  | TB positives: All active TB (Microbiologically confirmed, probable, and possible (n = 58)  TB negatives: All controls (no TB, no HIV) (n = 100) | | | | | | | |
| **Cut-off** | **Sensitivity (%)** | **95% CI** | **Specificity (%)** | **95% CI** | **Youden’s Index (J)** | **95% CI** | **PPV (%)** | **NPV (%)** |
| 0.010 | 100 | 0.93-1.00 | 2 | 0.0-0.07 | 0.02 | 0.00 – 0.05 | 22 | 100 |
| 0.020 | 100 | 0.93-1.00 | 13 | 0.07-0.20 | 0.13 | 0.06 – 0.20 | 25 | 100 |
| 0.030 | 100 | 0.93-1.00 | 27 | 0.19-0.36 | 0.27 | 0.18 – 0.36 | 27 | 100 |
| 0.040 | 100 | 0.92-1.00 | 40 | 0.30-0.49 | 0.40 | 0.30 – 0.50 | 31 | 100 |
| 0,050 | 100 | 0.92-0.99 | 52 | 0.42-0.61 | 0.52 | 0.42 – 0.62 | 37 | 99 |
| 0,060 | 100 | 0.92-0.99 | 63 | 0.53-0.71 | 0.63 | 0.54 – 0.72 | 45 | 98 |
| 0,070 | 98 | 0.90-0.99 | 75 | 0.65-0.82 | 0.73 | 0.64 – 0.82 | 50 | 97 |
| 0,080 | 96 | 0.88-0.99 | 79 | 0.70-0.85 | 0.75 | 0.67 – 0.83 | 57 | 96 |
| ***0,090*** | ***94*** | ***0.85-0.98*** | ***88*** | ***0.80-0.93*** | ***0.82*** | ***0.74 – 0.90*** | ***80*** | ***95*** |
| ***0,100*** | ***91*** | ***0.81-0.96*** | ***88*** | ***0.80-0.93*** | ***0.79*** | ***0.70 – 0.88*** | ***84*** | ***93*** |
| 0.115 | 82 | 0.71-0.90 | 88 | 0.80-0.93 | 0.70 | 0.59 – 0.81 | 84 | 93 |
| 0,125 | 79 | 0.67-0.87 | 88 | 0.80-0.93 | 0.67 | 0.56 – 0.78 | 84 | 92 |
| 0,150 | 74 | 0.61-0.83 | 96 | 0.80-0.96 | 0.70 | 0.59 – 0.81 | 88 | 90 |
| 0,200 | 58 | 0.45-0.70 | 93 | 0.86-0.96 | 0.51 | 0.39 – 0.63 | 93 | 90 |
| 0,225 | 55 | 0.42-0.67 | 99 | 0.94-0.99 | 0.54 | 0.42 – 0.66 | 93 | 90 |
| 0,250 | 53 | 0.40-0.65 | 100 | 0.94-0.99 | 0.53 | 0.41 – 0.65 | 95 | 88 |
| 0,300 | 50 | 0.37-0.62 | 100 | 0.96-1.00 | 0.50 | 0.37 – 0.63 | 96 | 77 |

**Supplementary Table 2: Inter-laboratory precision assessment of plasma Kynurenine and Tryptophan concentrations measured at NICD and Houston Laboratories**

| **Tryptophan** | | | | | | | | | | | | | | |
| --- | --- | --- | --- | --- | --- | --- | --- | --- | --- | --- | --- | --- | --- | --- |
|  | **NICD lab** | | | | | **Houston lab** | | | | |  |  |  | Inter-lab |
|  | *Day 1* | *Day 2* | *Day 3* | *Day 4* | *Day 5* | *Day 1* | *Day 2* | *Day 3* | *Day 4* | *Day 5* | Mean | SD | CV | %CV |
| CTL1 (µmol/L) | 50.382 | 51.225 | 50.118 | 51.006 | 53.391 | 61.175 | 54.445 | 59.999 | 57.506 | 59.003 | 54.825 | 4.261 | 0.078 | 6.171 |
| CTL2 (µmol/L) | 147.689 | 154.367 | 148.595 | 151.678 | 152.437 | 138.651 | 145.848 | 148.230 | 155.389 | 148.178 | 149.106 | 4.815 | 0.032 | 3.229 |
| **Kynurenine** | | | | | | | | | | | | | | |
|  | **NICD lab** | | | | | **Houston lab** | | | | |  |  |  |  |
| CTL1 (µmol/L) | 1.916 | 1.977 | 1.989 | 1.951 | 2.056 | 2.157 | 2.175 | 2.159 | 1.973 | 2.099 | 2.045 | 0.097 | 0.047 | 4.721 |
| CTL2 (µmol/L) | 9.694 | 9.694 | 9.780 | 10.517 | 9.756 | 8.568 | 9.283 | 9.587 | 8.817 | 9.809 | 9.551 | 0.549 | 0.057 | 5.749 |
| **K/T ratio** | | | | | | | | | | | | | | |
|  | **NICD lab** | | | | | **Houston lab** | | | | |  |  |  |  |
| CTL1 | 0.038 | 0.039 | 0.040 | 0.038 | 0.039 | 0.035 | 0.040 | 0.036 | 0.034 | 0.036 | 0.037 | 0.002 | 0.053 | 5.268 |
| CTL2 | 0.066 | 0.063 | 0.066 | 0.069 | 0.064 | 0.062 | 0.064 | 0.065 | 0.057 | 0.066 | 0.064 | 0.003 | 0.052 | 5.173 |

**Supplementary Figure 1**

**Supplementary figure 1***: Comparison of plasma Kynurenine/Tryptophan (K/T) ratios between microbiologically confirmed and clinically diagnosed TB cases in the Optimization and Validation Cohorts. In the optimization cohort, there were 11 microbiologically confirmed TB cases and 12 clinically diagnosed (unconfirmed) TB cases. In the validation cohort, there were 15 microbiologically confirmed TB cases and 20 clinically diagnosed (unconfirmed) TB cases. In the optimization cohort, microbiologically confirmed TB cases showed a trend toward higher K/T ratios than clinically diagnosed TB cases, although this difference did not reach statistical significance (P = 0.0792). In contrast, the validation cohort demonstrated a statistically significant elevation in K/T ratio among microbiologically confirmed TB cases compared with clinically diagnosed TB cases (P = 0.0290). The lines in the middle show the median.*
